# Supplementary material for: A Hierarchical Model to Predict Time of Flowering of Kiwifruit Using Weather Data and Budbreak Dynamics
Source: Plants (Basel). 2024 Aug 12;13(16):2231. doi: 10.3390/plants13162231 (PMC11359339; doi:10.3390/plants13162231)
Supplement: Supplementary file 1 [file plants-13-02231-s001.zip › plants-3108042-supplementary.pdf]

Supporting Materials:

Table S1. List of kiwifruit flowering data used.

	Study Year	Region	Cultivar	Orchard
1	2008	Te Puke	‘Hayward’	Orchard1
2	2008	Te Puke	‘Hayward’	Orchard2
3	2009	Te Puke	‘Hayward’	Orchard1
4	2009	Te Puke	‘Hayward’	Orchard2
5	2010	Kerikeri	‘Hayward’	Orchard3
6	2010	Te Puke	‘Hayward’	Orchard1
7	2010	Te Puke	‘Hayward’	Orchard2
8	2011	Kerikeri	‘Hayward’	Orchard3
9	2013	Kerikeri	‘Hayward’	Orchard4
10	2013	Te Puke	‘Zesy002’	Orchard5
11	2014	Kerikeri	‘Zesy002’	Orchard6
12	2014	Kerikeri	‘Zesy002’	Orchard4
13	2014	Kerikeri	‘Zesy002’	Orchard7
14	2014	Kerikeri	‘Zesy002’	Orchard8
15	2014	Kerikeri	‘Zesy002’	Orchard9
16	2014	Kerikeri	‘Hayward’	Orchard10
17	2014	Kerikeri	‘Hayward’	Orchard11
18	2014	Kerikeri	‘Hayward’	Orchard7
19	2014	Kerikeri	‘Hayward’	Orchard8
20	2014	Te Puke	‘Zesy002’	Orchard5
21	2015	Kerikeri	‘Zesy002’	Orchard4
22	2015	Kerikeri	‘Hayward’	Orchard4
23	2015	Te Puke	‘Hayward’	Orchard5
24	2016	Te Puke	‘Zesy002’	Orchard12
25	2016	Te Puke	‘Hayward’	Orchard13
26	2018	Kerikeri	‘Zesy002’	Orchard4
27	2018	Kerikeri	‘Hayward’	Orchard4
28	2018	Te Puke	‘Zesy002’	Orchard5
29	2018	Te Puke	‘Hayward’	Orchard5
30	2019	Kerikeri	‘Zesy002’	Orchard4
31	2019	Kerikeri	‘Hayward’	Orchard4
32	2020	Kerikeri	‘Zesy002’	Orchard14
33	2020	Kerikeri	‘Zesy002’	Orchard4
34	2020	Kerikeri	‘Hayward’	Orchard15
35	2020	Te Puke	‘Hayward’	Orchard16
36	2020	Te Puke	‘Hayward’	Orchard5
37	2021	Kerikeri	‘Zesy002’	Orchard14
38	2021	Kerikeri	‘Hayward’	Orchard17
39	2022	Kerikeri	‘Zesy002’	Orchard18

40	2022	Kerikeri	‘Hayward’	Orchard4
41	2022	Te Puke	‘Hayward’	Orchard19

Table S2. Model selection for classification models. Mean Akaike Information Criterion (AIC) and Bayesian Information Criterion (BIC) values of binomial, Poisson, and polynomial models in budbreak prediction.

Region	Cultivar	Binomial		Poisson		Polynomial	
		AIC	BIC	AIC	BIC	AIC	BIC
Kerikeri	‘Zesy002’	7.1	8.9	81.4	83.1	71.8	74.5
Kerikeri	‘Hayward’	9.7	11.2	54.1	55.5	37.8	40.0
Te Puke	‘Zesy002’	5.8	6.2	52.3	52.7	51.7	52.3
Te Puke	‘Hayward’	7.4	7.4	35.4	35.4	26.2	26.3

Table S3. The minimum kiwifruit first flowering day predicted by the selected model for 2020 to 2022 compared with the observations as the test data. The predictive variables included in the model were cultivar, predicted 5% budbreak date, daylength on 1 September.

Year	Region	Cultivar	Earliest date of flowering day			Range of first flowering	
			Predicted	Observed	Difference	Predicted	Observed (days)
2020	Kerikeri	‘Zesy002’	301.2	297.0	4.2	301.2-326.9	25.7
2020	Kerikeri	‘Hayward’	319.6	314.0	5.6	319.6-343.6	27.1
2020	Te Puke	‘Hayward’	315.9	316.0	-0.1	315.9-339.8	21.3
2021	Kerikeri	‘Zesy002’	303.2	302.0	1.2	303.2-328.9	22.7
2021	Kerikeri	‘Hayward’	323.8	312.0	11.8	323.8-347.8	33.3
2022	Kerikeri	‘Zesy002’	309.2	308.0	1.2	309.2-337.8	22.6
2022	Kerikeri	‘Hayward’	314.9	318.0	-3.1	314.9-354.3	18.7
2022	Te Puke	‘Hayward’	312.1	318.0	-5.9	312.1-344.8	30.0

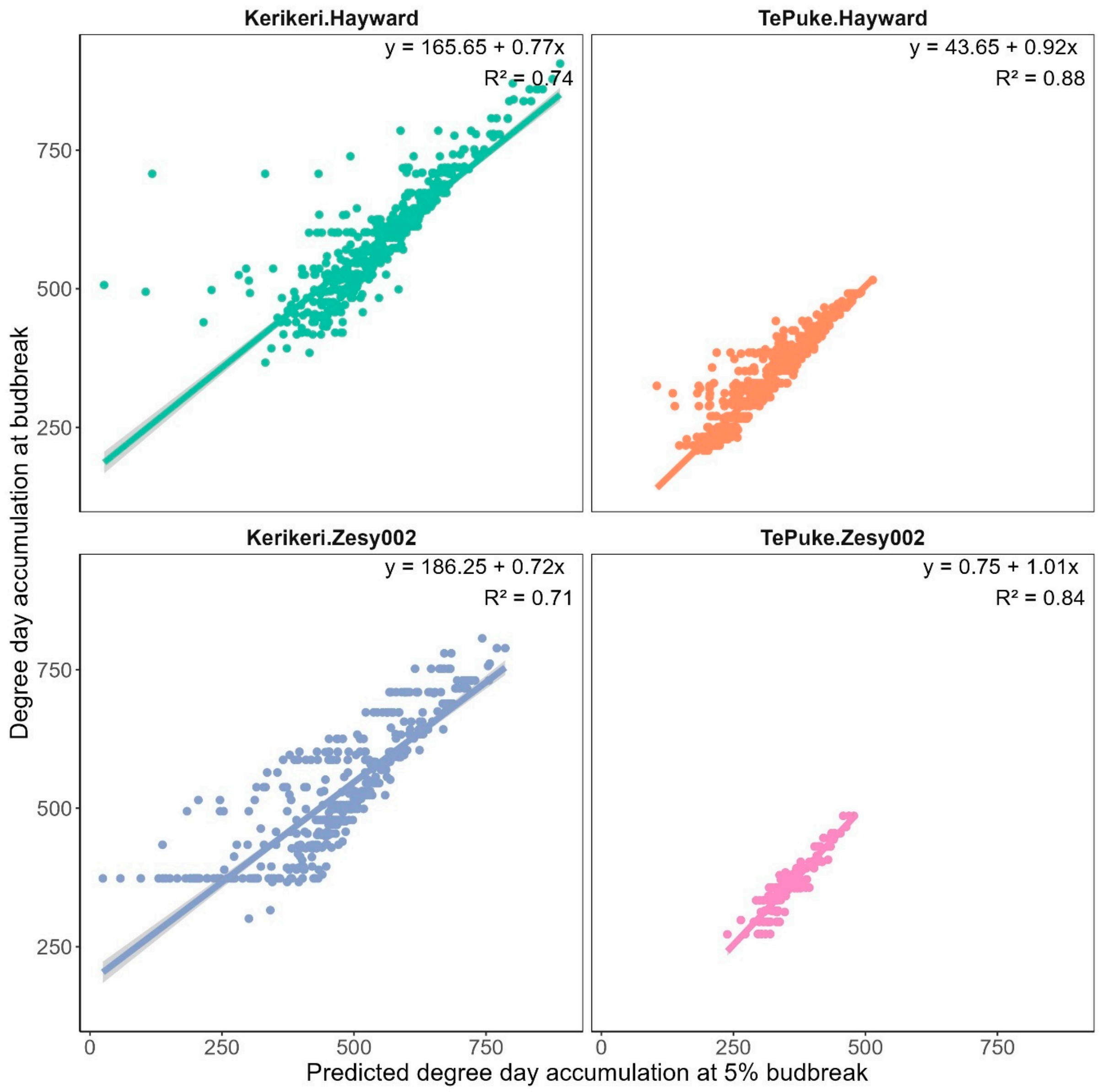

Figure S1. Predicted Degree Day Accumulation at 5% Budbreak and the observed day of the Year of the 5% budbreak from the kiwifruit budbreak count. The linear regression lines illustrate trends for different cultivars in the two study regions. The regression model parameters (slope, intercept) and the coefficient of determination (R^2) are displayed at the top right of each panel.

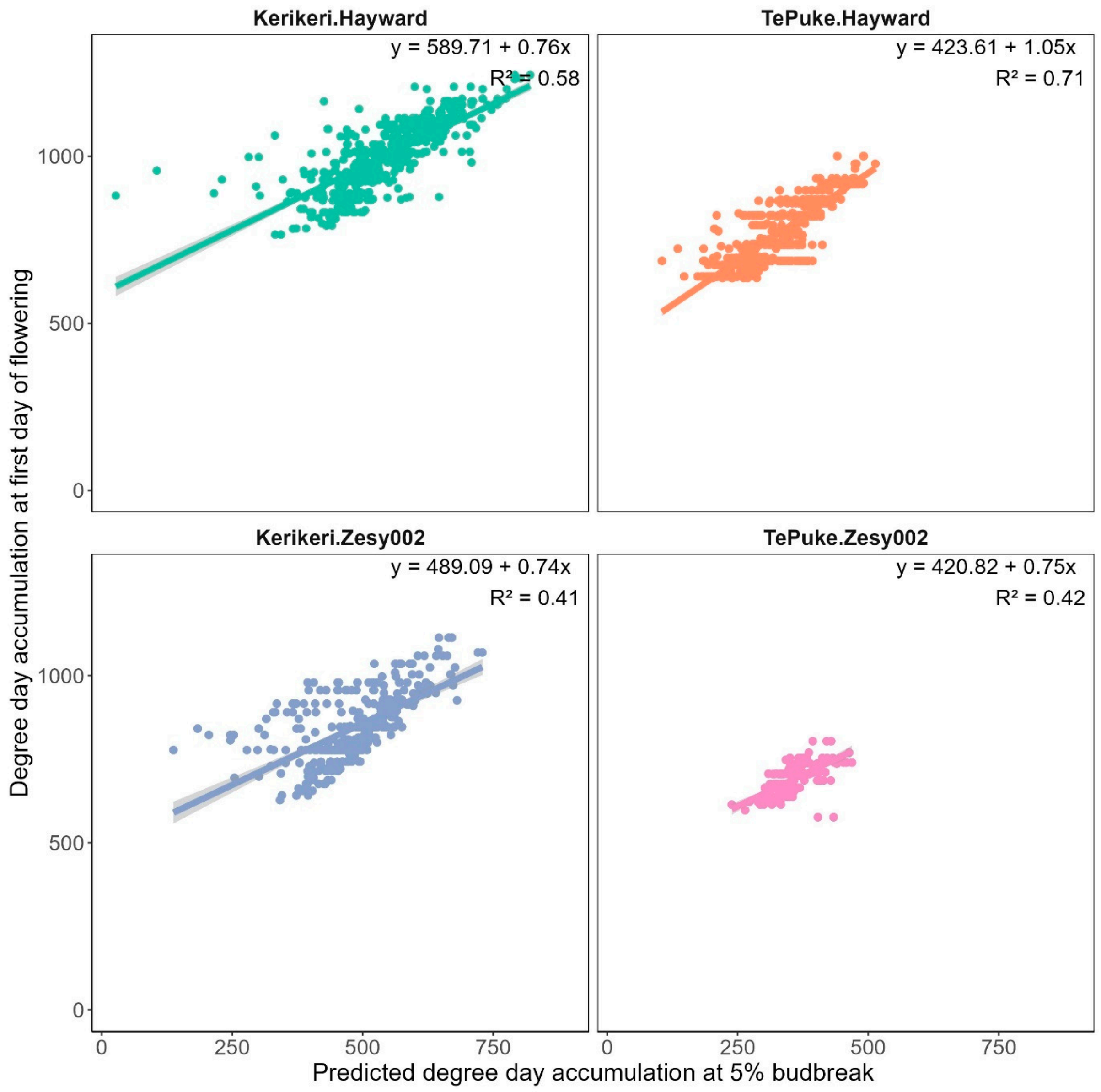

Figure S2. Predicted Degree Day Accumulation at 5% Budbreak and the observed day of the year of first flowering of kiwifruit. Each point represents the day of the year of first flowering plotted against the degree day accumulation at 5% budbreak, with linear regression lines illustrating trends for different cultivars in the two study regions. The regression model parameters (slope, intercept) and the coefficient of determination (R^2) are displayed at the top right of each panel.

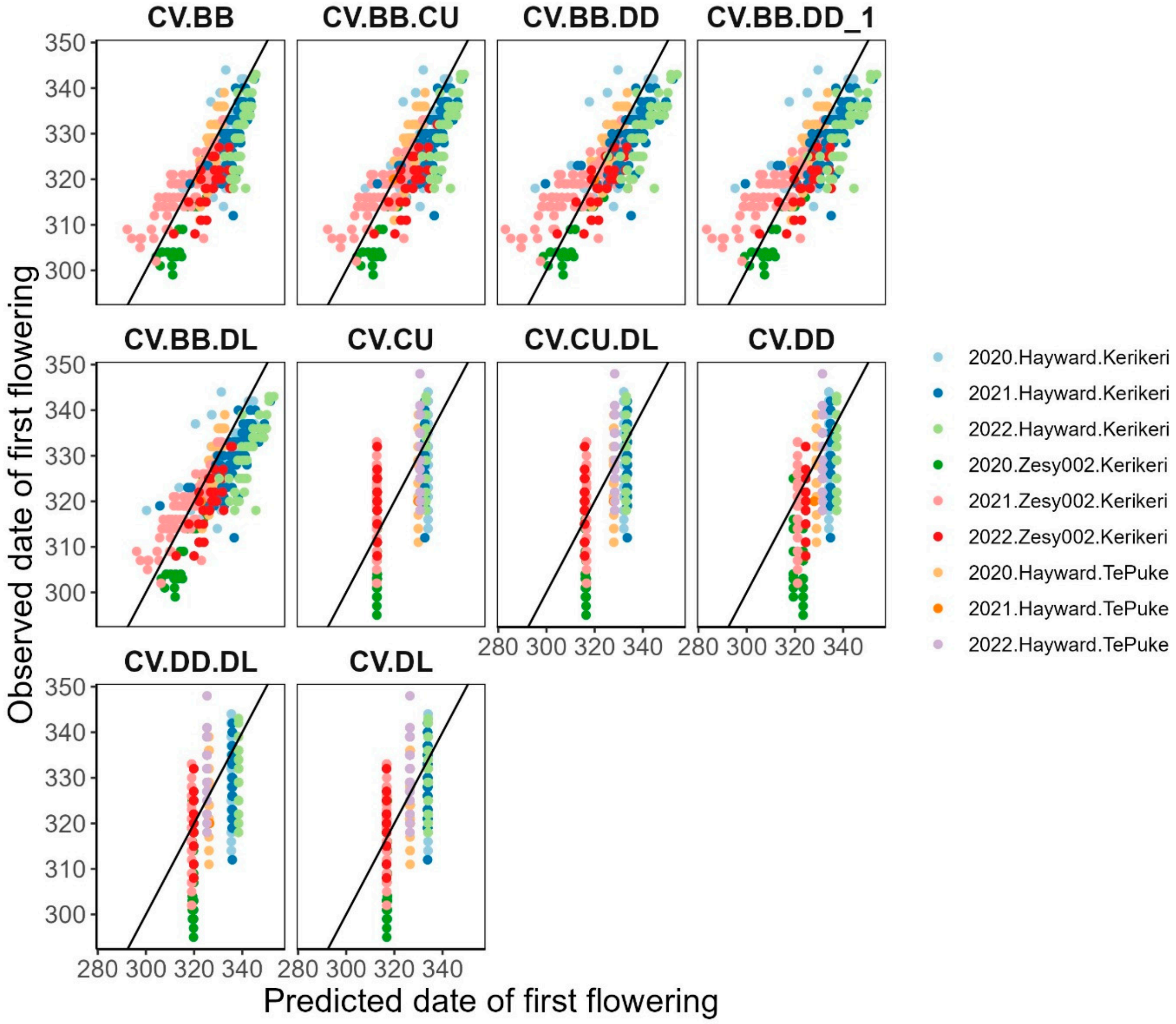

Figure S3. Observed day of first flowering for a kiwifruit vine versus predicted by the various combination of predictive variables. The names of the models on the top of each plot suggest the predictive variables that were included in the model. The abbreviations of the model names are as follows: CV = cultivar, BB = predicted 5% budbreak date, DD is degree day accumulation on 1 September, CU is chilling unit accumulation on 1 September, DL is daylength on 1 September. The solid line depicts a 1:1 relationship.

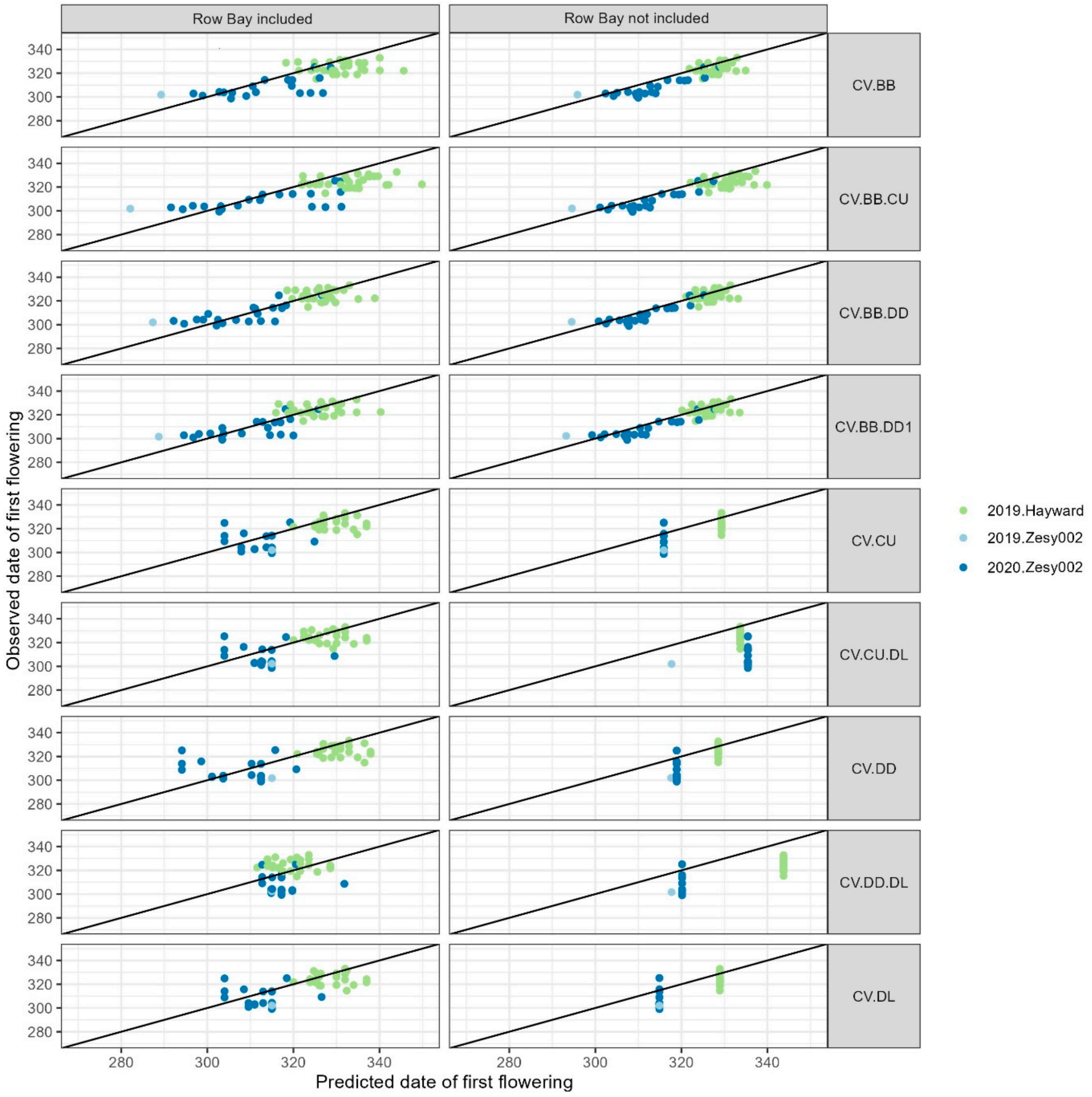

Figure S4. Observed day of first flowering for a kiwifruit vine versus predicted for control plants in orchards where row and bay position were known in the Kerikeri study orchard. CV = cultivar, BB = predicted 5% budbreak, DD is degree day accumulation on 1 September, CU is chilling unit accumulation on 1 September, DL is daylength. The solid line depicts a 1:1 relationship.
